# The expectations of transgender people in the face of their health-care access difficulties and how they can be overcome. A qualitative study in France

**DOI:** 10.1017/S1463423620000638

**Published:** 2020-12-16

**Authors:** Emmanuel Allory, Ellie Duval, Marion Caroff, Candan Kendir, Raphaël Magnan, Bernard Brau, Elinore Lapadu-Hargue, Sidonie Chhor

**Affiliations:** 1 Department of general practice, University of Rennes 1, F-35000 Rennes, France; 2 CIC (Clinical investigation centre) INSERM 1414, F-35000 Rennes, France; 3 École des hautes études en santé publique (EHESP), Saint-Denis, France; 4 Réseau Santé Trans association

**Keywords:** transgender persons, health services accessibility, health-care disparities, primary health-care, population health management

## Abstract

**Aim:**

Our objective was to explore the difficulties experienced by transgender people in accessing primary health-care services and their expectations towards primary care providers to improve their health-care access.

**Background:**

Because transgender people are exposed to many discriminations, their health-care access is particularly poor. Guidelines recommend greater involvement of primary care providers in the processes because of the accessibility feature of primary care services.

**Methods:**

A qualitative study using semi-directed interviews was conducted among 27 transgender people (February 2018 – August 2018). These voluntary participants were recruited through different means: local trans or LGBTI (lesbian, gay, bisexual, trans, and/or intersex) associations, primary care providers, and social networks. The data analysis was based on reflexive thematic analysis in an inductive approach.

**Findings:**

Difficulties in accessing health-care occurred at all the levels of the primary health-care system: primary care providers – transgender people interaction, access to the primary care team facility (starting with the secretariat), access to secondary care specialists, and continuity of care. Transgender people report ill-adapted health-care services as a result of gender-based identification in health-care settings. Their main expectation was depsychiatrization and self-determination. They supported mixed health network comprising primary care providers and transgender people with a coordinating role for the general practitioner. These expectations should be priorities to consider in our primary health-care system to improve access to health-care for transgender people.

## Introduction

Transgender people’s health is affected by numerous factors causing health disparities, including stigmatization, discrimination, pathologization, and social and economic marginalization (United Nations High Commissioner for Human Rights, 2011). Socio-legal policies vary from one country to another. While 95% of the Organisation for Economic Co-operation and Development (OECD)’s countries (including France) allow transgender people to change their gender marker in the civil registry, only 37% of them (including France) do not condition legal gender recognition on medical requirements (OECD, 2020). Health systems and settings are themselves among the causal factors of health disparities that are nevertheless avoidable, remediable, and unfair (Braveman, 2006; United Nations High Commissioner for Human Rights, 2011; Reisner *et al.*, 2016). Yet access to care of transgender people is inadequate due to several factors (*Transgender EuroStudy* 2008, | *ILGA-Europe*, 2008; Grant *et al.*, 2010; Cruz, 2014; Whitehead, Shaver and Stephenson, 2016; Wylie *et al.*, 2016).

Many transgender people delay, avoid, or refuse health-care as a result of past experiences in care settings (Grant *et al.*, 2010; Whitlock *et al.*, 2019). A national study in New Zealand on the health and well-being among transgender people showed that over a third of participants had avoided seeing a doctor because of disrespect or mistreatment concerns (‘Community Report – Counting Ourselves’, 2019). Those that decide to receive health-care travel three times as likely as cisgender people to finding a health-care center that they can trust (Whitehead *et al.*, 2016). Transgender people report high rates of mistreatment in health-care encounters, refusal of substandard care due to stigmatization, and medical providers’ lack of knowledge. They also mention difficulties related to hormone therapies and limited access to safe prescribing and follow-up for hormone therapy (Grant *et al.*, 2010; Kosenko *et al.*, 2013; Wylie *et al.*, 2016). On top of that, transgender people are at risk for several health problems and they need regular, comprehensive care (Feldman *et al.*, 2016; James *et al.*, 2016).

In the literature, many researchers have studied transgender people’s health in terms of specific diseases (Sanchez *et al.*, 2009; Asscheman *et al.*, 2011; Rotondi *et al.*, 2012; Blosnich *et al.*, 2014; Weinand and Safer, 2015; Brown and Jones, 2016; Seelman *et al.*, 2017). A recent global burden of disease review demonstrated that the most common research topics are mental health diseases, sexual and reproductive health, and substance use (Reisner *et al.*, 2016). Some authors have explored whether hormone therapy should be initiated in primary care, or the willingness of primary care providers to monitor hormone therapy for transgender populations (Wylie *et al.*, 2016; Shires *et al.*, 2018). Besides this, guidelines have been published for the best inclusive practices in primary care for transgender people, including comprehensive preventive care services (Coleman *et al.*, 2012; Edmiston *et al.*, 2016; Wylie *et al.*, 2016; Aitken, 2017; Nisly *et al.*, 2018; Whitlock *et al.*, 2019).

Implicating primary care providers in the care of transgender people is recommended as one of the strategies to improve care in this population (Whitlock *et al.*, 2019). Hence, as part of the primary care team, the general practitioner (GP) has a vital role in improving access to primary care services for transgender people, and in regaining their trust in the health-care services by building an on-going trustful relationship during consultations (WONCA Europe 2002; Robles *et al.*, 2016; Campbell *et al.*, 2018). However, the health system organization and patient pathways for transgender people still differ between countries and within them (*Transgender EuroStudy 2008 | ILGA-Europe*, 2008).

In France, there are two points of entry into the health-care system for transgender people. They can either access care via primary care (through a GP) or via hospital care (by way of the *Société française d’études et de prise en charge de la transidentité – SOFECT*). Both are covered by the national health insurance (known as *Assurance Maladie*) but there are major differences between those two pathways.

In the hospital one, the person can have access to a team without systematic referral by a GP. Differing from the World Professional Association for Transgender Health standards of care (WPATH) which promote a flexible approach (Coleman *et al.*, 2012), the SOFECT procedure involves six steps with systematic psychiatric evaluation and collective decision at every step (*Prise en charge globale et médicale dans le domaine de la transsexualité*, 2020). Medical and surgical treatments are mostly considered as treatment of a psychiatric illness. However, to our knowledge, the hospital trajectory generally entails long waiting times and is considered to be more paternalist in decision-making.

The person can also access to care via a primary care pathway, with the support of a mixed network of health-care providers and local trans associations, in a way close to the WPATH standards of care (Coleman *et al.*, 2012; Askevis-Leherpeux *et al.*, 2019; *Reseau sante trans*, 2020). The mixed network can give support to the health-care providers, with opportunities for exchange on practices and improvement of their medical skills. The person makes an appointment with the GP, and a reactive, personalized evaluation is conducted. Transgender people are considered by GPs like any other people with a medical request (Askevis-Leherpeux *et al.*, 2019). In this trajectory, to our knowledge, access to care seems easier, without a systematic psychiatric evaluation, and without long waiting lists. Decisions are shared with the transgender individual. Hence, ensuring the access of transgender people to primary care services is important, not only for their specific problems but also to maintain their health and well-being via screening, vaccination etc. as in the general population (Edmiston *et al.*, 2016).

In order to improve access to primary care and to optimize the care of transgender people, the answer to the difficulties experienced by transgender people in accessing care and the identification of what they expect from primary care professionals is needed.

The main objective of the present study was to explore the difficulties of transgender people in accessing primary care. The secondary objective was to explore the expectations of transgender people towards the health-care system and primary care professionals.

## Methods

### Study design

A qualitative study was carried out by performing semi-structured individual interviews among transgender individuals from western France. This study was approved by the Rennes University Hospital ethics committee on 12 March 2018 (Number 18.20).

### Recruitment

Participants were recruited in three ways. Firstly through two local trans associations in Rennes: Ouest Trans and ISKIS. These associations used their social networks to spread the information (facebook^®^, twitter^®^). The other recruitment source was through primary care providers consulting with transgender people. Ouest Trans is an association which aims to be a self-help self-support trans association, based in Rennes, and also covering all the western part of France. Its mission is to fight transgender phobia and its consequences, such as the isolation of transgender people (Ouest trans, 2020). ISKIS is the LGBTI center in Rennes, and a member of the national federation of LGBTI centers which combat sexism, male domination, and control over bodies and sexualities. A special group of transgender people is part of it (Iskis, 2020).

For the recruitment of the participants, leaflets, including general information about the study, were distributed in the premises of associations and in the waiting rooms of primary care providers. Individuals were able to take away the leaflets and distribute them in their own networks. Interested individuals were invited to contact the research team via an email address and phone numbers of the researchers given on the leaflet or in the announcement in the social media. The inclusion criteria for the study were being 18 years old or over and identifying oneself under the terms trans/transgender/trans-identity/transsexual/in a process of transition. We purposively selected participants among individuals contacted to ensure variation in gender, age, transition starting year, socio-economic group, and place of residence (MacDougall and Fudge, 2001).

### Interviews and data collection

The interviews took place from February 2018 to August 2018 (duration: 16–125 min, median: 55 min). The choice of the location and the time of the interview were left to the participants, to make them feel more comfortable (home, public place like a café, public park, university). The interviews involved one interviewer and one transgender individual. Written informed consent was obtained from each interviewee before the interview. The two interviewers (ED, MC) introduced themselves as female cisgender, residents in family medicine. They were previously trained in qualitative methods with a senior researcher (EA). The interviewers informed interviewees that they were conducting this work in an academic perspective (thesis). The interviewers did not know any of the interviewees.

The interview guide was developed on the basis of a literature review performed at the beginning of the study and discussed with all the co-authors (Supplementary table 1). The final version was pilot-tested with one male transgender person and primary care providers involved in transgender care (two female GPs, one female endocrinologist–gynecologist). The first warm-up question invited the respondents to talk about their last contact with their GP. The second question aimed to explore the reason why the person chose their GP. The following question talked about the role of the GP in their care pathway. The fourth question investigated the difficulties that the transgender respondents had experienced in the course of their health-care pathway. Then, we asked them what could be their expectations to improve their access to health-care. Another question explored their point of view on the existing mixed network in the area. Finally, the last question collected the different socio-economic elements defined in the study protocol. All the interviews were digitally recorded and the verbatim was transcribed, using Microsoft Word software. There was no feedback to participants for comment or correction and no repeat interview was conducted. Field notes were taken throughout and kept in a logbook by the interviewers.

### Data analysis

The analysis followed the reflexive thematic analysis theory, in an inductive approach (Braun and Clarke, 2019). Two researchers (ED, MC) manually and independently coded the interviews implementing the six phases of the thematic analysis: familiarization with the data, generation of initial codes, search for themes, review of themes, definition and naming of themes, and drafting of the report (Braun *et al.*, 2006). The coding framework was discussed regularly in the research group (BB, EA, ED, ELH, MC, RM, SC) to establish a consensus and resolve disagreements. The difficulties were classified depending on the level of the health-care system, following the Jan de Maeseneer classification (Maeseneer *et al.*, 2014). Nano level involve the relation between the patient and the health-care provider. Micro level involves the primary care team. Meso level involve the patient’s health-care pathway and his potential interaction with secondary or tertiary care. Finally macro level involves the organization of the health-care system.

## Results

A total of 30 people contacted the interviewers. Only 27 of them were included in the study. Two did not respond to the email interview appointment proposal, and one did not call back after a postponed interview. The characteristics of the participants are presented in Table 1. Age was divided in four categories, and we had 12 participants in category 18–30 years, 10 in the 30–40 group, 4 in the 40–50 group, and 1 in the 50–60 group.

**Table 1.** Characteristics of the transgender respondents and interviews

Participant identification	Gender self-definition	Age (years)	Transition starting year	Recruitment mode	Socio-economic group*	Living place	Volunteering in trans association	Length of interview (min)
P1	M	50–60	2016	Family doctor	Industrial skilled employee	Urban	Yes	70
P2	M	30–40	2012	Family doctor	Clerical and skilled service employees	Rural	Yes	51
P3	F	40–50	2005	Family doctor	Technicians and associated professional employees	Urban	Yes	75
P4	M	30–40	2013	Social networks	Other non-employed persons	Urban	No	84
P5	M	40–50	2012	Social networks	Technicians and associated professional employees	Rural	No	69
P6	F	18–30	2017	Association	Other non-employed persons	Urban	Yes	52
P7	M	18–30	2017	Association	Other non-employed persons	Urban	Yes	49
P8	M	30–40	2013	Association	Professionals	Rural	No	62
P9	F	18–30	2018	Family doctor	Other non-employed persons	Urban	Yes	50
P10	M	18–30	2015	Family doctor	Clerical and skilled service employees	Urban	No	63
P11	M	18–30	2018	Association	Other non-employed persons	Urban	Yes	50
P12	F	18–30	2016	Association	Professionals	Urban	Yes	58
P13	F	40–50	2018	Social networks	Industrial skilled employee	Rural	No	60
P14	F	30–40	2017	Family doctor	Professionals	Urban	No	45
P15	M	18–30	2008	Association	Professionals	Rural	Yes	40
P16	M	30–40	2015	Family doctor	Clerical and skilled service employees	Urban	Yes	83
P17	F	40–50	2006	Family doctor	Other non-employed persons	Urban	No	19
P18	F	30–40	1998	Family doctor	Industrial skilled employee	Urban	No	16
P19	M	18–30	2018	Family doctor	Other non-employed persons	Urban	Yes	125
P20	F	30–40	2017	Family doctor	Other non-employed persons	Urban	No	90
P21	M	18–30	2017	Association	Other non-employed persons	Urban	Yes	28
P22	F	30–40	2017	Social networks	Industrial skilled employee	Urban	Yes	16
P23	M	18–30	2013	Social networks	Other non-employed persons	Urban	No	66
P24	Non binary	30–40	2018	Association	Other non-employed persons	Urban	No	65
P25	M	18–30	2017	Association	Other non-employed persons	Rural	Yes	36
P26	F	18–30	2015	Social networks	Other non-employed persons	Urban	Yes	22
P27	F	30–40	2014	Social networks	Professionals	Urban	Yes	29

P = Participant; M = Male; F = Female.

* Socio-economic groups are described based on the European Socio-economic Groups (ESEG).

We present below the difficulties of transgender people at different levels of health-care : nano, micro, meso and macro levels (Maeseneer *et al.*, 2014). In terms of what the respondents expected from primary care professionals, four major themes emerged from the interviews.

### Difficulties in accessing primary health-care

As a preamble to the difficulties encountered with the health-care system, transgender people often expressed individual difficulties in recognition of their gender identity by those around them, creating situations of stress. Additional pressure was experienced when approaching the health-care system because of the challenges of a successful ‘passing’, meaning being perceived as the gender they wish to, in relation to considerable hetero-normativity representations in their entourage and society.

*But at first, I used to get stressed every time I went into a waiting room. Now I think I have taken it upon myself. As I am feeling good about myself, I don’t care what people think. (P1)*

The impact of a difficult transition could lead to vulnerabilities for transgender people at different levels. Firstly, in terms of psychological health, with isolation from friends and family, and secondly, in terms of professional aspects, sometimes the cessation of studies or work, leading to financial insecurity and dependence on a third party.

*Actually, the trans issue made a lot of people break off with me. At first, for example, my family. That is complicated, and now we’ve broken off, it’s over. (P5)*

### A primary care provider – transgender people relationship to build – nano level

A first difficulty in accessing care concerned the anxiety of the first visit to a new caregiver. The difficulty to know or not if the caregiver will have knowledge about transgender care was a difficulty, leading some respondents to withdraw from care. When accessing care, some spoke of discriminatory attitudes by certain caregivers, either consciously or unconsciously, usually due to a lack of knowledge about the health of transgender people.

*She [*GP*] told me that she saw a show on TV about trans people and once she saw a trans person in consultations ” So I saw a trans man, he was really a man, he wasn’t like you, he really looked like a man (laughs) he had a beard and everything”. (P5)*

The failure to seek permission for the clinical examination was another issue that makes respondents question the GP’s approach. In addition, the lack of genuine consideration for the transgender people as partners in care was cited.

*I found it rather embarrassing that she was checking my underpants. I know that medically it could be useful, but she could have at least explained it to me. (P19)*

### Complex access to the primary care team – micro level

Transgender respondents reported gender-inappropriate reception in primary care services. The first professionals they met were the secretaries, who were not always aware how to receive transgender people. Then, the experience of the waiting room was also perceived as frightening, particularly with the looks of the other patients in the room. Finally, the doctor’s call for the next patient in the waiting room was described as the most anxiety-provoking moment in the consultation process, with a risk of experiencing an ‘outing’ that means disclosing the gender identity of the person without that person’s consent. All of these elements in the primary care team’s attitudes can lead transgender people to give up consulting a doctor or to experience mental difficulties in accessing care.

*Before reaching the doctor, there are the secretaries, the waiting room, the telephone…For me it’s what was the most complicated in the end. (P2)*

As a consequence, transgender people form their own networks of trusted primary care providers and do not hesitate to travel several hundred kilometres to obtain quality care. The participants frequently mentioned the need to take a day off school or work. The fragility and saturation of these networks, frequently experienced by transgender communities, were also underlined.

*Actually, the thing is that as soon as I leave the network that I have formed, I find myself back to square one, faced with people’s incomprehension, that’s when it is difficult and eventually blocks access to care. (P16)*

### Specific conditions to access a surgical team and out-of-hours medical services – meso level

Access to hospital surgical teams, particularly SOFECT, was considered difficult by trans people, who considered the inclusion criteria of their protocol too normative. Thus, conditioning the initiation of the transition process in the hospitals to a systematic psychiatric evaluation was considered unnecessary, so that they sometimes abandoned the transition process.

*I think it is a shame that we are forced, for certain types of operations, for certain doctors, to spend two years in mental health care. In fact, I do not see the point, we know who we are and we do not need someone to validate who we are. (P25)*

On the other hand, the interviewees did not appreciate the gender normativity applied by health-care providers in binary manner.

*It was actually a lot about what you look like, like you had to have long hair to be a trans woman. (P6)*

Another mandatory step was presented as particularly difficult: the so-called ‘real-life experience’. This step consists in asking people to live in the desired gender in order to assess their motivation for the transition, imposing a lifestyle change when the physical transformations had not yet taken place.

*The “real-life experience” consists in allowing a period of months or years to force the person to be, socially, the gender they want. Even if on paper, you are still the original sex, […] to show that you are motivated. So it is not necessarily easy, depending on how you look and how you are perceived from the outside. (P4)*

Beside the transition process, transgender people also reported anticipatory anxiety related to access to emergency care at the hospital. Fear of a potentially difficult experience with an unfamiliar team was expressed.

*For example, what worries me is that I might have to go to an emergency department that I did not choose, with health care professionals that I won’t know. (P16)*

### A health-care system that is not adapted to transgender people – macro level

Even if the organization of the social welfare system is independent from gender, some transgender people experienced difficulties obtaining reimbursements as a result of local administrative practices. For instance, gynecological procedures like cervical smears could not be performed because of the need for gender congruence in the procedure. Other elements evoked in particular were the requirement by certain health insurances to have a certificate from a psychiatrist providing for 100% reimbursement of care by the health insurance system.

*In health insurance companies, they categorize medical acts between men and women, a man cannot have a gynaecological consultation for example. Therefore, I found myself at one point off the list. (*P6)

### Expectations concerning the health-care system and primary care professionals

The expectations of the transgender people surveyed revolved around four dimensions: main ethical principles (self-determination, depsychiatrization), a genuine health-care partnership, a central place for the GP in the healthcare pathway, and a respectful attitude without financial barriers to access health-care.

### Main ethical principles: self-determination and depsychiatrization

The interviewees called on two principles. The first was self-determination in their gender identity. They meant that it is not the professionals who decide on the gender of the person, but the transgender people themselves. This demand was accompanied by a demand for a broader consideration of gender, which they felt was too binary (woman or man). Secondly, the transgender respondents wanted a depsychiatrization of transgender identity by health professionals, entailing a necessary re-evaluation of the recommendations at national level.

*Having a doctor who does not systematically consider transgender as a psychiatric disease or automatically medicalize my trans-identity is very important. (P25)*

### A real patient – primary care provider partnership

A real partnership of care with caregivers was desired by the transgender respondents. Recognition of transgender peoples’ experiences and scientific knowledge on the part of the health-care professional was desired. In this relationship, the health-care provider’s interpersonal skills were particularly valued. The lack of medical scientific expertise on transgender people was fully accepted so long as the professional recognized it and expressed willingness to learn.

*That is where Dr. X really is an ally, because she understands the request. She listens to her patient, because I tell her what to write, because I know what the court wants to hear. (P16)*

### A central place for GPs in a health network in partnership with the trans associations

The place of GPs in the health-care pathway was considered central. First of all, as part of the transition process, people wanted them to be able to initiate and follow-up treatments. The flexibility of their practice, their accessibility, and their global approach were valued for reasons other than transgender identity. Their role as coordinators of the pathway was not particularly emphasized.

*So finally between a GP that I can see quite easily and an endocrinologist who will potentially be more expensive and less accessible […] I prefer the GP because he is also a doctor I can see outside my transition*. *(*P9)

Transgender people valued the existence of a mixed health network of caregivers and transgender associations, and identified it as a factor facilitating their access to transition. The interviewees felt confident with GPs who were members of the network. Within this network, special attention was paid to the balance of power between caregivers and transgender people and to joint management.

*For the GP I went to see, I know there was a subject she was not too familiar with, so she asked another person who was part of the network. So, it’s true that it’s fairly reassuring to see that links are also being created between doctors. It creates more trust in the doctor. (P9)*

### A respectful health-care system without financial barriers

The transgender respondents called for a respectful acceptance of their identity in health facilities, and particularly the absence of misgendering when arriving at the reception with the secretaries. Training for the health-care staff was also called for in order to improve their reception.

*The first step would be for the secretariat be trained to deal with trans people and to ask what name to use at the first meeting. The secretariat should know about the fact that the first name on the ID card is not necessarily the same as the name given for the appointment. (P7)*

The other element facilitating access to care would be the existence of a fee waiver in advance, which could be included in all-inclusive care by the health insurance (Affection Longue Durée – ALD)

*I had difficulties to access to care and the ALD triggers everything else. So that is the first stone turned before anything else happens. (P2)*

## Discussion

In a life course often made up of breakdowns (family, friends, and work) (United Nations High Commissioner for Human Rights, 2011), sometimes complicated by precariousness, the transgender people interviewed described difficulties in accessing care at all levels of the health system (Maeseneer *et al.*, 2014): in the health-care provider–patient relationship (nano); on the level of the primary care team (micro); on the level of access to secondary care (meso), and on the level of legislative and administrative aspects of the health system (macro).

Expectations revolved around four dimensions: main ethical principles, which were self-determination and depsychiatrization of transgender-identity; a real partnership between health-care providers and transgender people; a central place for the GP in the healthcare pathway; and finally, an easier access to care from an administrative and financial point of view.

## Strengths and limitations

The strength of our study is that it focuses for the first time, to our knowledge, on what transgender people experience in access to primary care and what they expect from primary care providers. Another strength is the number of people interviewed. Considering the discrimination they are experiencing in the health-care system, the agreement by transgender people to be interviewed by two residents in family medicine could have been difficult. The involvement of the trans associations in the recruitment probably helped transgender people approached to be trustful towards the interviewers.

This study do have some limitations. First, we did not find any person without primary care follow-up in our sample. Transgender people without follow-up in primary care could have enriched and diversified the results of the study. However, these results can be considered in the light of accessibility to primary care (World Health Organization, 2008). Secondly, even though we interviewed 27 transgender people, we did not find any results on access to treatment via non-standard channels (through internet, via trans friends, etc.) (IGAS, 2011). Two reasons already reported in the French literature might explain this limitation. The status of the interviewers who introduced themselves as medical students could have negative influence on the discourse of the interviewees. In addition, the professional structure created in the western part of France where the study was conducted may improve health-care access for transgender people.

## Comparison with the existing literature

### Difficulties in access to care: a discriminated population, a major issue in primary care

In our results, the notion of relational discrimination by health-care providers emerged – including secretariats. This is fostered by the existence of misrepresentations about transgender-identity and generates practices that are sometimes inappropriate towards transgender people (United Nations High Commissioner for Human Rights, 2011; Suess Schwend, 2020). Experiences of discrimination are also reported because of the organisation of the health system. For example, some protocols require transgender people to undergo 2 years of psychiatric follow-up and real-life experience before any hormone therapy instatement. This exposes them to numerous discriminations (Bujon and Dourlens, 2012; Coleman *et al.*, 2012; Abramovich and Cleverley, 2018). The recurrence of stressful situations is described in the literature as ‘*minority stress*’ and corresponds to the chronic stress faced by members of stigmatized minority groups. Several studies on perceived discrimination and health have also shown that this chronic stress can ultimately have a negative impact on physical and mental health, and access to care among the people concerned (Pascoe and Smart Richman, 2009; Testa *et al.*, 2017). Considering the different theoretical frameworks that exist on health determinants, access to health-care is a major determinant of the health of these populations (Émond *et al.*, 2010; World Health Organization and the United Nations Children’s Fund, 2018). Adapting primary care systems to welcome transgender populations in an appropriate and caring manner is therefore a major issue in the health of transgender people.

Participants discussed different solutions to improve their access to care. Firstly, the interviewees proposed a central place for the GP. By way of their particular characteristics (comprehensive approach, accessibility), GPs enable a reduction in the levels of morbidity and mortality among transgender people (Reisner *et al.*, 2016; Wylie *et al.*, 2016; Aitken, 2017). In many health-care systems, GPs are the primary prescribers of hormone treatments (Sansfaçon *et al.*, 2018; Askevis-Leherpeux *et al.*, 2019).

### Expectations: self-determination and depsychiatrization

Transgender respondents expressed a desire for a caring relationship based on a true partnership, underpinned by respect for self-determination as recognized by WPATH and other studies. (*Standards of Care - WPATH World Professional Association for Transgender Health*, 2012; Pomey *et al.*, 2015; Eyssel *et al.*, 2017; Nieder *et al.*, 2017; Lampalzer *et al.*, 2019). However, the training of health-care professionals aims to educate them to recognize normal pathologies, according to rigorous scientific reasoning (Sironi, 2011). Self-determination of identity by the transgender person overturns both this scientific reasoning and the prerogatives of the health-care provider in his or her relationship with the transgender person (Castel, 2019). In order to take the patient’s perspective more fully into account, some care venues call on the notion of the ‘*obviousness of transidentity*’, as a starting point for the accompaniment of transgender people by the primary care team (Askevis-Leherpeux *et al.*, 2019).

Another strong expectation was the demand for depsychiatrization of the transgender-identity pathway.(Askevis-Leherpeux *et al.*, 2019). Most respondents questioned the systematic and mandatory nature of psychiatric evaluations. They wanted psychiatric follow-up to be adapted to the needs and requests of the persons concerned. One of the first arguments in favor of depsychiatrization is that the main characteristics of mental disorders among transgender people result more from social rejection and violence than from transgender-identity per se (Robles *et al.*, 2016; Campbell *et al.*, 2018; Askevis-Leherpeux *et al.*, 2019). In addition, this systematic psychiatrization of the health pathway contributes to precariousness in access to rights and the creation of barriers to accessing appropriate health-care (Robles *et al.*, 2016). For example, the term ‘therapeutic shield’ is sometimes used to describe the control exercised by psychiatrists over transgender people’s access to care by way of the issue of certificates (Espineira, 2011).

Further to this, the participants wanted health-care providers to be trained to deal with transgender people, and valued the network structure. Several authors stress the importance of training health-care providers to accompany transgender people, whether in initial training (Wylie *et al.*, 2016) or in continuing education in the context of health-care pathways. This should include culturally appropriate training, resources, and services, in partnership with associations (Ng, 2016; ‘Community Report – Counting Ourselves’, 2019).

## Implications for research and/or practice

Our findings lead to recommendations for health professionals and health policy makers. Health-care professionals should:

- Consider the plurality of gender identities: the objectification of discrimination in our study should encourage health-care professionals to undertake training about the specific dimensions that the transgender public is questioning: particularly gender identity. The traditional binary view needs to evolve towards a greater consideration of different identities.
- Engage a true care partnership: in the perspective of individual self-determination, the evolution of the health-care provider – patient relationship towards a closer partnership will make it possible to recognize the autonomy of transgender people and to accompany them in the best possible way.
- Adopt a global approach to care: each caregiver should use the bio-psycho-social approach (Freeman and McWhinney, 2016) when accompanying transgender people, taking into account the impact of social determinants on access to care (financial barriers in particular).

According to our results, public authorities should implement a depsychiatrization of transgender-identity and widely adopt the standards of the WPATH.

In terms of research, the identification of difficulties in access to primary care could be pursued through intervention studies testing the recommendations on access to care for transgender people. Moreover, studies can be pursued about the perspectives of primary health-care provider about the difficulties of transgender people to access to health-care.

## Conclusion

This study highlights the difficulties in accessing care for transgender people at all levels of health-care. Considering the expectations of this population towards all the actors of primary care and integrating them into the health-care system should be a priority. One of the first steps should be to re-evaluate the professional recommendations in partnership with transgender people, including primary care providers as coordinators and first actors in the health-care pathway.

## Supplementary material

For supplementary material accompanying this paper visit https://doi.org/10.1017/S1463423620000638. click here to view supplementary material
