# Supplementary material for: The expectations of transgender people in the face of their health-care access difficulties and how they can be overcome. A qualitative study in France
Source: Prim Health Care Res Dev. 2020 Dec 16;21:e62. doi: 10.1017/S1463423620000638 (PMC7801925; doi:10.1017/S1463423620000638)
Supplement: Supplementary file 1 [file S1463423620000638sup001.docx]

Supplementary table 1: Individual interview guide

| Nº | Interview questionsº |
| --- | --- |
| 1 | When was your last consultation with your general practitioner?  (Could you describe the last time you consulted a general practitioner from the starting point to the end of the consultation) |
| 2 | What characteristics are you looking for in your general practitioner?  (What were the reasons you chose your general practitioner?)  (What do you think is important to consider in the choice of a general practitioner?) |
| 3 | What do you think is the role of the general practitioner in your health care pathway?  (Can you describe the different steps of your health care pathway in the context of your transition and the role that your general practitioner played?) |
| 4 | In this pathway, did you have difficulties in accessing to care? What were they?  (What were the discriminations you experienced in accessing health care?)  (Among your friends or community, to your knowledge, what kind of difficulties do trans people experience in accessing the health care system?) |
| 5 | If you could imagine a better health-care pathway for yourself, what would you change?  (What would be your expectations from the health care providers you are consulting?) |
| 6 | A mixed network of health care providers and trans people exists in the district, what would you expect from it? |
| 7 | Collection of the characteristics of the respondents |

Sentences in parentheses were suggestion for rewording in case of difficulties for interviewees in understanding the first one.

Supplementary table 2. The difficulties of transgender people in accessing primary health care

| **Themes** | **Verbatim** |
| --- | --- |
| A primary care provider – transgender people relationship to build - nano level | ‘He gave me a prescription with my female birth name and it's unthinkable for me to have that, even my employer put my male first name (...) I asked him to do it again for the physiotherapy and he took his pen and he crossed out the "Madame" and said 'you're not going to make a fuss' and he threw the paper at me.’EE5  ‘Except that the problem is just that, in the end, I'm the one who punishes myself because I'm afraid, so I postpone appointments, which adds to the physical and psychological deterioration of trans people.’ EE12 |
| Complex access to the primary care team – micro level | ‘I am in (name of the city), I can find doctors, but what's it like in the country? Well, it's impossible!’ EE12  ‘I was referred to family planning because there is a good psychologist. But they told me, ‘He's overbooked, he's not taking new patients’.’ EE10  ‘I missed a day of classes (...) I had catch a train, I had to spend 75 euros for the day to get my testosterone (...) I had to give up a whole day and the money, which could clearly have been used for food or to get treatment’ EM5 |
| Specific conditions to access a surgical team and out-of-hours medical services – meso level | ‘I have always actually believed, because of that damn SOFECT, that trans people are just people with mental problems, people who are all depressed and end up committing suicide.’ EE8  ‘They wanted to take care of the perineal area, so at the time I was not reluctant to have a hysterectomy because I don't want to carry a child so I don't need it, the uterus. [...] But for me it was very clear from the beginning, I didn't want sexual reassignment surgery, I wasn't interested in it…’ EE4 |
| A health care system that is not adapted to transgender people – macro level | ‘I have had a few depressive episodes because it's been very hard to survive over that time. Because living with an identity that is not right also means hiding your ID. And therefore not showing up, when you're asked ‘Madam, can I have your ID card’, I answer ‘no’’ EE6  ‘Well administrative papers are a problem because I have the 2 (meaning "female") in front and I can't have anything else but the 2 in front until I make my change in gender status, which I intend to do but which only comes at the end of the transition.’EE5 |

Supplementary table 3. The expectations from the health care system and primary care professionals

| **Themes** | **Verbatim** |
| --- | --- |
| Main ethical principles: self-determination and depsychiatrisation | ‘It is very rare to meet doctors like Dr. V. who told me ‘*I would have prescribed the treatment even without a psychiatrist's certificate*’ because she says it is not an obligation, it is not considered a mental pathology, there's no obligation to go through a psychiatrist.’ EE13  ‘When they asked me what I wanted to be called. I told them "EM10". They said, ‘Mr or Mrs?’ I said Mr. […]. I thought that was good. They are looking out for their patients, and their well-being, their choices. That is what it is all about. They respect choices.’ EM 10 |
| A real patient – primary care provider partnership | ‘Just the ability to say when she didn't know, I thought it was really great, and really rare in fact.’ EM1  ‘And so, when we met, we talked more about associations than anything else. I told her that if she met other trans people she could bring them to the ‘Ouest Trans’ Association and the CGLBT. I also gave her an information booklet for doctors.’EE7 |
| A central place for GPs in a health network in partnership with the trans associations | ‘With my GP it was ‘Are you OK?’, ‘Do you have any relatives?’, and ‘Are you with someone?’... And actually I think it is good when the doctors ask if you are ok and not "explain to me, tell me your whole life history.’EE7  ‘It is true that we are a little afraid of the associations gathering health professionals who are interesting to work towards transgender people. But then again, I know the people who are part of ReST. Sometimes it is scary. We are thinking: aren't they going to get ahead of us and stop listening to us? Knowing that you work with trans people makes me feel a lot better…’ EE11 |
| A respectful healthcare system without financial barriers | ‘At the GP's consultation, you hear a lot of the ‘Mr and Mrs. What would be nice would be to just say the first name or last name.’ EE8 |
